# Development and Evaluation of a Novel Chimeric Genotype VII Newcastle Disease Vaccine: Overcoming Maternal Antibody Interference and Spray Administration

**DOI:** 10.3390/vetsci11110532

**Published:** 2024-11-01

**Authors:** Xiaoquan Wang, Yao Yao, Wenhao Yang, Xiaolong Lu, Ruyi Gao, Kaituo Liu, Yu Chen, Min Gu, Jiao Hu, Shunlin Hu, Xiufan Liu, Xiaowen Liu

**Affiliations:** 1 Key Laboratory of Avian Bioproducts Development, Ministry of Agriculture and Rural Affairs, School of Veterinary Medicine, Yangzhou University, Yangzhou 225009, China; wxq@yzu.edu.cn (X.W.); yaoyao@hjmd.cc (Y.Y.); 008465@yzu.edu.cn (W.Y.); 008328@yzu.edu.cn (X.L.); 007622@yzu.edu.cn (R.G.); biochenyu@hotmail.com (Y.C.); gumin@yzu.edu.cn (M.G.); hijiao@yzu.edu.cn (J.H.); slhu@yzu.edu.cn (S.H.); xfliu@yzu.edu.cn (X.L.); 2 Jiangsu Co-Innovation Center for Prevention and Control of Important Animal Infectious Diseases and Zoonosis, Yangzhou University, Yangzhou 225009, China; liukaituo@yzu.edu.cn; 3 Jiangsu Key Laboratory of Zoonosis, Yangzhou University, Yangzhou 225009, China; 4 Joint International Research Laboratory of Agriculture and Agri-Product Safety, The Ministry of Education of China, Yangzhou University, Yangzhou 225009, China

**Keywords:** NDV, chimeric vaccine, spray administration

## Abstract

Newcastle disease virus (NDV) poses a significant threat to the poultry industry, with the emergence of genotype VII NDV leading to extensive outbreaks and economic losses. Vaccination is the primary means of controlling NDV, but the presence of maternal antibodies (MDAs) can interfere with the immunological effect of live virus vaccines. Thus, we constructed a chimeric NDV live virus vaccine, LX-OAI4S, by replacing the extracellular regions of the F and HN genes of the NDV LX strain with the corresponding regions of the A-VII vaccine strain. The chimeric vaccine LX-OAI4S demonstrated high genetic stability, good safety, and strong reproductive capacity in chicken embryos. The LX-OAI4S vaccine induced rapid antibody production in specific pathogen-free (SPF) and commercial chickens via the intranasal and intraocular (IN/IO) routes, with hemagglutination inhibition (HI) antibody titers reaching 4.71 ± 1.03 log_2_ at 7 days post-vaccination (dpv), significantly higher than those of the two classical vaccine strains La Sota and VG/GA. The LX-OAI4S vaccine group provided effective protection against the challenge of genotype VII NDV virulent strain JS2/06 and inhibited viral shedding. When administered via spray, the LX-OAI4S vaccine elicited high systemic immunity against NDV in both SPF and commercial chickens, effectively protecting against clinical disease and reducing viral shedding. The chickens were exposed to high-dose vaccine for spray vaccination, and no adverse reactions were observed after vaccination. Despite the presence of anti-NDV MDAs in chickens, the NDV-specific antibody titers were significantly greater in the vaccinated groups than in the unvaccinated group. The vaccine exhibited high immunogenicity and the potential to overcome maternal antibody interference. The LX-OAI4S vaccine is a promising candidate for an ND vaccine. Its administration via spray can effectively prevent the occurrence of ND, making it a valuable tool for the poultry industry.

## Simple Summary

Newcastle disease is a devastating virus that affects poultry, causing illness, death, and significant economic losses. Current live vaccines struggle to protect against the most common type, genotype VII, and can be hindered by maternal antibodies. We have developed a new vaccine called LX-OAI4S by combining two strains with different genotypes, bringing together the advantages of both strains. This hybrid vaccine offers strong protection against genotype VII Newcastle disease and works well even when chickens have maternal antibodies. Importantly, it can be easily administered through a spray, making it a promising tool for poultry farmers to prevent outbreaks and protect their flocks.

## 1. Introduction

Newcastle disease virus (NDV) is currently causing a widespread and enduring pandemic, impacting extensive geographical regions ranging from Central and Eastern Europe to Southeast Asia. This situation is leading to significant economic losses for the poultry industry. NDV not only causes high morbidity and mortality rates in poultry but also poses challenges in vaccination and disease control due to its high transmissibility and variability. Despite the availability of various vaccines for controlling NDV, the continuous evolution of the virus and the development of new vaccines remain areas of active research [1].

Most Class II NDV strains, based on the fusion cleavage sites within the fusion protein, are considered to be virulent and have been responsible for the majority of ND outbreaks reported to date. Additionally, Class II NDV exhibits high genetic diversity, which can be categorized into at least 20 genotypes [2]. Notably, the genotype VII of Class II NDV led to the fourth Newcastle disease pandemic, which is still affecting regions in Asia, Africa, Europe, and South America [3]. In China, genotype VII NDV is the predominant circulating genotype, causing the majority of ND outbreaks in poultry [4].

The current prevalence of NDV genotype VII worldwide highlights its widespread transmission and variation across multiple countries and regions. In Asia, particularly Vietnam and Indonesia, the prevalence and variation in genotype VII NDV has garnered significant attention. In northern Vietnam, a highly pathogenic subgenotype VII.2 has been detected, sharing similar genetic sequences with isolates from Vietnam, China, and South Africa, indicating the geographical distribution and genetic diversity of NDV [5]. Indonesia, the global second-largest poultry producer after China, has suffered significant economic losses due to the prevalence of NDV in the local poultry industry. Despite the implementation of vaccination control programs in Indonesia, NDV outbreaks still occur periodically. The predominant NDV genotype circulating in Indonesia is genotype VII, followed by genotype VI and I. In 2019, 10 strains of NDV were isolated from commercial poultry farms in South Sulawesi, Indonesia, which had the pathogenic marker sequence 112R-R-Q-K-R-F117 in the F protein cleavage site. The identity of these strains with NDV genotype VII.2 ranged from 87.82% to 97.96% [6]. Furthermore, genotype VII NDV is the most prevalent NDV genotype in Africa, having been found in 15 countries. The distribution of genotype VII NDV varies across different regions in Africa, being most common in North Africa and South Africa, for example, in Libya [7], Egypt [8], Mozambique [9], South Africa, Botswana [10], and Zambia [11]. In summary, genotype VII, represented by the two subgenotypes VII.1.1 and VII.2, is the main circulating genotype in Africa due to its association with multiple outbreaks of ND in chickens [12].

For the current poultry industry, in addition to good biosecurity measures, vaccination is the primary measure for controlling the spread of NDV in chicken flocks. Previously, commercial ND vaccines mainly belonged to genotypes I (Ulster, QV4) and II (La sota, B1, VG/GA). However, mismatches between the vaccines and circulating wild-type strains can lead to inadequate immunity. Although vaccination usually prevents clinical disease and mortality associated with field NDV strains, it has a limited effect in preventing infection or virus shedding [13,14]. Minimizing the virus shedding by vaccinated chickens is a key focus in the management and control of ND vaccines [15]. Selecting a vaccine that corresponds to the genotype of the prevalent strain not only diminishes the incidence of disease within flocks but also diminishes viral shedding and transmission. The inactivated vaccine genotype VII NDV (A-VII) is compatible with the prevalent genotype VII NDV strains in China [16]. The A-VII vaccine was extensively commercialized in the poultry sector in 2014. Subsequently, the isolation rate of genotype VII NDV has progressively declined and is currently no longer detected in the commercial poultry industry [17]. This has made a significant contribution to the prevention and control of NDV in China and provided rich experience for the prevention and control of NDV in other regions of the world. The primary mode of immunization for inactivated vaccines involves injection, a procedure that induces considerable stress in chickens and that is both time-consuming and labor-intensive. With the continual growth of the poultry industry, there is a growing demand for the development of vaccines that are compatible with more convenient immunization techniques, such as spraying. Currently available ND vaccines do not offer a spray delivery option. ND live vaccines suitable for spray application have been the subject of intense research [18,19,20,21,22]. Nonetheless, ND live vaccines are prone to interference from maternal antibodies, which can impair the seroconversion of antibodies following vaccination [23,24]. Research has involved the replacement of the F and HN genes of NDV with the homologous genes from other avian paramyxoviruses, resulting in the generation of novel chimeric NDVs. These recombinant viruses have demonstrated the ability to elicit a robust immune response in the presence of maternal antibodies [25,26].

In this study, we utilized the genotype I NDV LX strain as a vector to develop a recombinant live vaccine. This was achieved by substituting the extracellular regions of its F and HN genes with the equivalent regions from the genotype VII NDV A-VII strain. This modification aims to copy the prevalent strains in the world while also ensuring low virulence and minimizing the impact of maternal antibodies.

## 2. Materials and Methods

### 2.1. Ethics Statement

All animal experiments were conducted in strict accordance with the guidelines outlined in the Guide for the Care and Use of Laboratory Animals by the Ministry of Science and Technology of the People’s Republic of China. The Yangzhou University Administrative Committee for Laboratory Animals (permission number: 202203151) approved all of the animal studies according to the guidelines of the Administrative Measures of Jiangsu Province on laboratory Animals of Jiangsu Administrative Committee of Laboratory Animals.

### 2.2. Cells, Viruses, Eggs, and Animals

Chicken embryo fibroblast (DF-1) cells were sourced from the China center for ATCC and cultured in Dulbecco’s Modified Eagle’s Medium (DMEM) supplemented with 10% fetal bovine serum, 100 U/mL penicillin, and 100 µg/mL streptomycin (Gibco, New Zeeland). Specific pathogen-free (SPF) eggs and chickens were purchased from Beijing Boehringer Ingelheim Vital Biotechnology Co., Ltd. (Beijing, China). The NDV genotype I avirulent strain LX was used as a vaccine vector in this study [27]. The La Sota vaccine, widely used globally as either a live or inactivated vaccine, was utilized in this study as a control live vaccine for the immunization challenge experiments. For the challenge phase of the vaccination experiment, the wild-type NDV strain JS2/06 was utilized. All viruses were obtained from Yangzhou University (Yangzhou, Jiangsu, China); the virus stocks were propagated and titrated in 9–10-day-old SPF embryonated chicken eggs.

### 2.3. Construction of the Chimeric NDV cDNA Clone pLX-OAI4S

**Figure 1:**
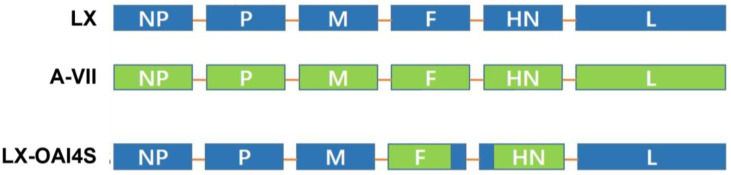
Schematic description of the structure of the LX-OAI4S. The ectodomain of the F and HN gene of the NDV LX strain was replaced by that of the vaccine strain A-VII. Blue color represents genes derived from the LX strain; green color represents genes derived from the A-VII strain.

The construction of a chimeric cDNA clone, designated pLX-OAI4S, was based on the infectious cDNA clone of the genotype I NDV attenuated strain LX (pLX). In this construct, the extracellular regions of the F and HN genes from the LX strain were substituted with the extracellular region of the F and HN genes from the inactivated ND vaccine strain A-VII (released in China in 2014), respectively. The sequence encoding the protease cleavage site of the F0 protein of vaccine strain A-VII was altered through PCR mutagenesis, as previously described [16]. In summary, the ectodomains of the F and HN gene of NDV were amplified via overlap extension PCR using specific primers. The amplified ectodomain of the F and HN fragments were inserted into Blunt-Zero using the restriction enzymes PshA I/Not I, respectively, to generate the pFHNs. Then, the pFHNs was digested with Spe I and Sac II to obtain the chimeric LX/I4 fragment, which was purified and cloned into Spe I/Sac II-digested pLX to generate pLX-OAI4S (Figure 1).

### 2.4. Virus Rescue

The rescue of the recombinant virus followed a procedure outlined in earlier studies [16]. Specifically, the full-length cDNA clone pLX-OAI4S was co-transfected with three helper plasmids derived from ZJ1 according to previously described methods [28]. After three days, the supernatants and cell monolayers from the transfected cultures were collected and used to inoculate the allantoic cavities of 9–10-day-old embryonated chicken eggs for virus rescue. To confirm the successful virus rescue and determine viral titers, these viral stocks were used to infect embryonated chicken eggs, and hemagglutination (HA) tests were conducted. Furthermore, RNA was extracted from the passaged viruses and subjected to reverse transcription polymerase chain reaction (RT-PCR) and sequencing analyses to assess the genomic stability of the LX-OAI4S construct.

### 2.5. Virus Titration and Pathogenicity Assessment

Chimeric viruses were characterized using standard HA assays and 50% tissue culture infectious dose (TCID50) assays performed on DF-1 cells. Additionally, the 50% egg infectious dose (EID50) assay was conducted using 9-day-old SPF chicken embryos [29]. To assess the pathogenicity of the recombinant viruses, the standard mean death time (MDT) and intracerebral pathogenicity index (ICPI) tests were performed [30].

### 2.6. Animal Experiments

**Figure 2:**
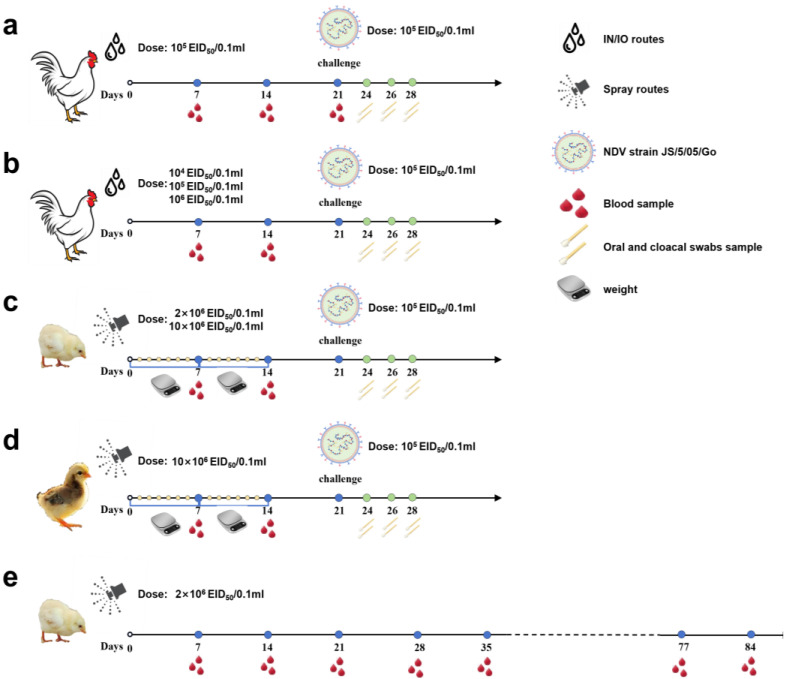
Flow chart of animal experiment implementation. (**a**) Three-week-old specific pathogen-free (SPF) chickens were vaccinated with recombinant virus strain LX-OAI4S, vaccine strain La Sota, VG/GA, and PBS, respectively, via the intranasal and intraocular (IN/IO) routes with a dosage of 10^6^ EID_50_/0.1 mL. Blood samples were collected on 7, 14, and 21 dpv to separate serum. Chickens were challenged with 10^5^ EID_50_/0.1 mL of NDV strain JS2/06 via the IN/IO route at 21 dpv. Oral and cloacal swabs were collected from birds to evaluate viral shedding at 3, 5, and 7 dpc. (**b**) Three-week-old SPF chickens were vaccinated with recombinant virus strain LX-OAI4S via the IN/IO routes with a dosage of 10^4^ EID_50_/0.1 mL, 10^5^ EID_50_/0.1 mL, and 10^6^ EID_50_/0.1 mL, respectively. Blood samples were collected on 7, 14, and 21 dpv to separate serum. Chickens were challenged with 10^5^ EID_50_/0.1 mL of NDV strain JS2/06 via the IN/IO route at 21 dpv. Oral and cloacal swabs were collected from birds to evaluate viral shedding at 3, 5, and 7 dpc. (**c**) One-day-old SPF chickens were vaccinated with recombinant virus strain LX-OAI4S, strain LX-OAI4S, La Sota, and VG/GA, respectively, via the spray routes with a dosage of 2 × 10^6^ EID_50_/0.1 mL and 10 × 10^6^ EID_50_/0.1 mL, respectively. The weight of chickens was continuously detected for 14 days after vaccination. Blood samples were collected on 7 and 14 dpv to separate serum. Chickens were challenged with 10^5^ EID_50_/0.1 mL of NDV strain JS2/06 via the IN/IO route at 21 dpv. Oral and cloacal swabs were collected from birds to evaluate viral shedding at 3, 5, and 7 dpc. (**d**) One-day-old commercial chickens were vaccinated with recombinant virus strain LX-OAI4S, La Sota, and VG/GA, respectively, via the spray routes with a dosage of 10 × 10^6^ EID_50_/0.1 mL. The weight of the chickens was continuously detected for 14 days after vaccination. Blood samples were collected on 7 and 14 dpv to separate serum. Chickens were challenged with 10^5^ EID_50_/0.1 mL of NDV strain JS2/06 via the IN/IO route at 21 dpv. Oral and cloacal swabs were collected from birds to evaluate viral shedding at 3, 5, and 7 dpc. (**e**) One-day-old SPF chickens were vaccinated with recombinant virus strain LX-OAI4S via the spray routes with a dosage of 2 × 10^6^ EID_50_/0.1 mL. Blood samples were collected every 7 days until day 84.

All animal experiments were conducted in the Animal Biosafety Level 3 animal facility of Yangzhou University. SPF chickens or commercial chickens were randomly grouped to evaluate the protective effect against the virulent NDV challenge (Figure 2). For the intranasal and intraocular (IN/IO) route vaccination, each chick received 100 μL of the indicated viruses or phosphate-buffered saline (PBS; pH 7.4). For spray vaccination, chicks were vaccinated following a standard protocol, using a vaccine spraying machine for administration (Xin Sheng Yuan, Shandong, China) with a droplet size of 140–180 μm, 16 mL/spray, and one spray for 100 chickens’ inoculation volume. Chicks were allowed to remain for at least 20 min after spraying to enhance the effect of preening. All groups of birds were monitored daily for clinical signs and blood samples were collected at 7, 14, and 21 days post-vaccination (dpv). At 21 dpv, chicks for further challenge protection experiments were challenged with NDV virulent strain JS2/06 via the IN/IO route. Morbidity and mortality were recorded for 14 days post-challenge (dpc).

### 2.7. Serology Test

Following immunization, blood was collected weekly and the antibody levels in the serum were quantified via a hemagglutination inhibition (HI) assay as described. Briefly, chicken serum was serially diluted two-fold with PBS and incubated with four HA units (HAUs) of the NDV antigen for 15–30 min at 37 °C, followed by incubation with 1% chicken red blood cells in 96-well V-bottom plates.

### 2.8. Virus Shedding in the in Tracheal and Cloacal Swabs of NDV

Oral and cloacal swabs were obtained from the chickens at 3, 5, and 7 dpc to assess viral shedding of the challenge virus. Virus recovery was evaluated using SPF eggs, as previously described [31]. In brief, each swab was inoculated into at least three 9-day-old SPF eggs through the allantoic cavity. NDV viral particles in the allantoic fluid of the inoculated eggs were detected using HI assays with an NDV-specific antibody.

### 2.9. Histology

Chickens were humanely euthanized at the specified time points following NDV infection. Lung, trachea, spleen and thymus tissues were collected and fixed with 4% formaldehyde, then embedded in paraffin. For histopathological examination, tissues were stained using the standard hematoxylin and eosin (H&E) method.

### 2.10. Statistics

Statistical analysis was performed using GraphPad Prism 5 software (GraphPad Software Inc., La Jolla, CA, USA). For comparing two groups, *p*-values were calculated using Student’s *t*-tests (two-tailed). For comparing more than two groups, one-way ANOVAs followed by Tukey’s test were used. A probability (*p*) value of <0.05 was deemed statistically significant.

## 3. Results

### 3.1. Development of a Chimeric ND Vaccine

To calculate the yield of the LX-OAI4S virus, we measured the EID_50_/0.1 mL and the HAUs in SPF eggs, as well as the TCID_50_/0.1 mL in cells cultured for up to 10 passages (Table 1). At the initial passage, the HA titer of LX-OAI4S was 8.5 log_2_, rising to 9.0 log_2_ by passage 10. The EID_50_ of LX-OAI4S was 8.5 log_10_/0.1 mL at passage 1, and 9.0 log_10_/0.1 mL at passage 10. At passage 1, 5, and 10, the MDT of LX-OAI4S was more than 120 h, and the ICPI was 0. At the fifth and the tenth passage, high TCID_50_ titers of LX-OAI4S were confirmed in the DF-1 cell (7.5 log_10_/0.1 mL).

### 3.2. Protective Efficacy of Recombinant ND Vaccine in SPF Chickens

**Figure 3:**
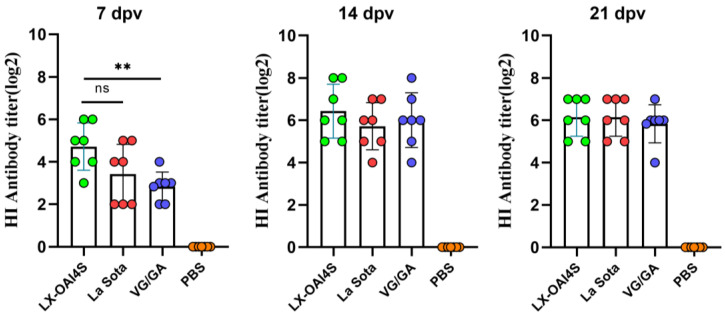
Hemagglutination inhibition (HI) antibody titers induced in chickens by ND vaccines. Specific pathogen-free (SPF) chickens were randomly divided into four groups (n = 7) and vaccinated with recombinant virus strain LX-OAI4S, vaccine strain La Sota, VG/GA, and PBS, respectively. Each chicken was vaccinated via the intranasal and intraocular (IN/IO) routes with a dosage of 106 EID50/0.1 mL. The PBS control group was also established. The status of each chicken was observed after vaccination, and blood samples were collected on 7, 14, and 21 dpv to separate serum. Green, red, blue, and orange colors represent the HI potency of the LX-OAI4S, La Sota, VG/GA, and PBS groups, respectively. The HI antibodies were then measured using the corresponding vaccine strains. Statistical analyses were performed by one-way ANOVA. ** *p* < 0.01; ns, not significant.

Chickens were vaccinated with LX-OAI4S by IN/IO routes to evaluate immune protective effects, while commercial vaccines Lasota and VG/GA were selected as positive controls. After vaccination, a continuous observation of 3 weeks showed that all immunized chickens had normal feeding and mental status. The results showed that at 7 dpv, the mean HI antibody titer in the LX-OAI4S group was 4.71 ± 1.03 log_2_, which was 1 log_2_ titer higher than that in the La Sota group with no significant difference, and 2 log_2_ titers significantly higher than that in the VG/GA group. At 14 dpv, the HI antibody titer in the LX-OAI4S group reached 6.43 ± 1.18 log_2_, which was 0.7 and 0.4 titers higher than that in the La Sota and VG/GA groups, respectively. At 21 dpv, the average antibody level in the LX-OAI4S group was higher than that in the other two vaccine strain groups (Figure 3). There was no significant difference among the three groups at 14 and 21 dpv.

To evaluate the protective efficacy of the vaccines, the chickens were challenged with NDV at 21 dpv. After the challenge, all chickens in the PBS control group died at 5 dpc, while none of the vaccinated groups showed clinical signs. In the LX-OAI4S group, no virus shedding was detected in the oral and cloacal swab samples at any time point after the challenge. In the La Sota group, two positive samples for virus shedding were found in the oral (2/7) and cloacal (2/7) samples at 3 dpc. In the VG/GA group, virus shedding was also detected in the oral and cloacal samples at 3 dpc, with virus shedding rates of 43% (3/7) and 14% (1/7), respectively (Table 2).

**Figure 4:**
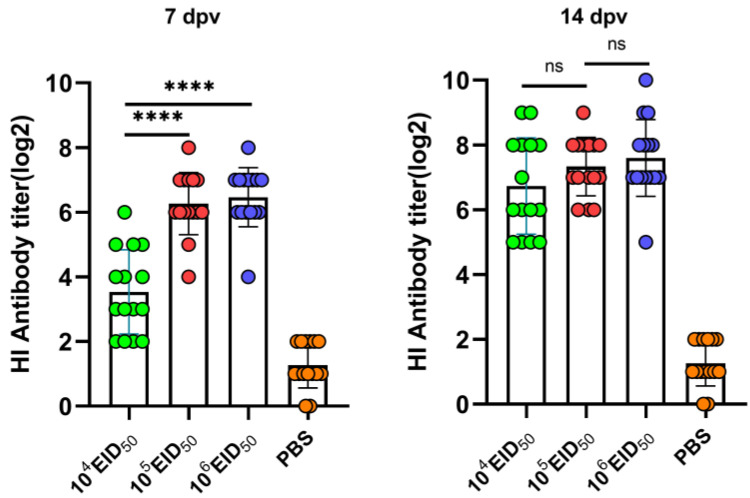
Hemagglutination inhibition (HI) antibody titers induced in chickens by LX-OAI4S with different doses. Specific pathogen-free (SPF) chickens were randomly divided into four groups (n = 15) and vaccinated with recombinant virus strain LX-OAI4S via the IN/IO routes with dosages of 104 EID50/0.1 mL, 105 EID50/0.1 mL, and 106 EID50/0.1 mL, respectively. The PBS control group was also established. The status of each chicken was observed after vaccination, and blood samples were collected on 7, 14, and 21 dpv to separate serum. Green, red, blue, and orange colors represent the HI potency of the LX-OAI4S, La Sota, VG/GA, and PBS groups, respectively. The HI antibodies were then measured using the corresponding vaccine strains. Statistical analyses were performed by one-way ANOVA. **** *p* < 0.0001; ns, not significant.

We further determined the minimum vaccination dose of LX-OAI4S and similarly assessed the HI antibody response after vaccination in chickens, as well as the mortality and virus shedding after NDV challenge. The HI antibody titer in the 10^4^ EID_50_ dose group reached 3.50 ± 1.26 log_2_ at 7 dpv, which was significantly lower than the titers in the 10^5^ EID_50_ and 10^6^ EID_50_ dose groups, which were 6.30 ± 0.93 and 6.53 ± 0.88, respectively. However, at 14 dpv, the HI antibody titer in the 10^4^ EID_50_ dose group reached 6.67 ± 1.44, which, although slightly lower than the 10^5^ EID_50_ and 10^6^ EID_50_ dose groups, showed no significant difference (Figure 4). Vaccination was followed by a challenge 14 days later, resulting in the death of all chickens in the PBS control group. In contrast, the LX-OAI4S vaccine groups immunized with different doses did not show clinical signs and all provided 100% protection. Swab samples were collected and tested for virus shedding by inoculating SPF chicken embryos. The results showed that only one chicken in both the 10^4^ EID_50_ and 10^5^ EID_50_ dose groups shed the virus from the oral route at 3 dpc with no subsequent shedding. In the 10^5^ EID_50_ dose group, no virus shedding was observed in the oral and cloaca at 3, 5, and 7 dpc (Table 3).

### 3.3. Protective Efficacy of Recombinant LX-OAI4S Vaccine via Spray Route in SPF Chickens

**Figure 5:**
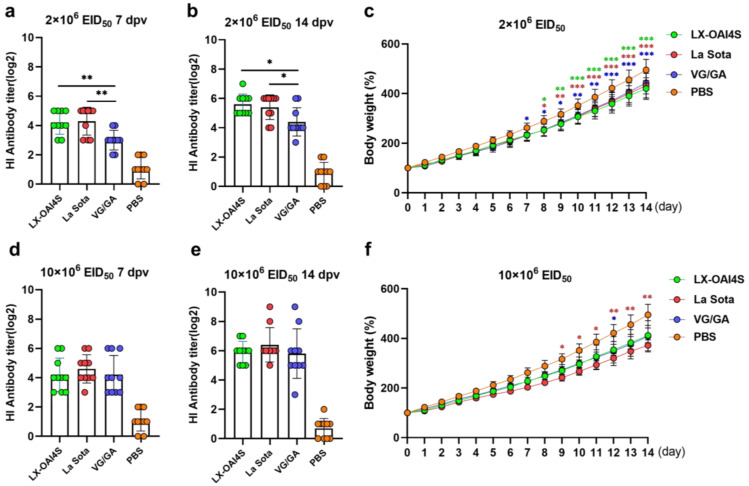
The HI antibody response and body weight changes in SPF chickens vaccinated with ND vaccine via the spray route. One-day-old specific pathogen-free (SPF) chickens were randomly divided into four groups (*n* = 10) and vaccinated with recombinant virus strain LX-OAI4S, La Sota, and VG/GA via the spray route, respectively. The PBS control group was also established. Two vaccination doses of the vaccines were used, 2 × 10^6^ EID_50_/0.1 mL (**a**,**b**) and 10 × 10^6^ EID_50_/0.1 mL (**d**,**e**). The status of each chicken was observed after vaccination, and blood samples were collected on 7 and 14 dpv to separate serum. The HI antibodies were then measured using the corresponding vaccine strains. Statistical analyses were performed by one-way ANOVA. * *p* < 0.05; ** *p* < 0.01; *** *p* < 0.001. The weight of chickens was continuously detected for 14 days after vaccination (**c**,**f**). The *p*-values shown for the comparison between the vaccinated group and the PBS group (**c**). The *p*-values shown for the comparison between the LX-OAI4S group and the La Sota or VG/GA group (**f**). Statistical analyses were performed by multiple *t*-tests. * *p* < 0.05; ** *p* < 0.01; *** *p* < 0.001.

To investigate the possibility of large-scale use in chicken flocks, we evaluated the immunoprotective efficacy of the spray administration of LX-OAI4S. The commercial vaccines VG/GA and La Sota served as positive control groups. Two vaccination doses of the vaccines were used, 2 × 10^6^ EID_50_/0.1 mL and 10 × 10^6^ EID_50_/0.1 mL. The purpose of the low-dose group is to evaluate the immunoprotective efficacy of the vaccine, while the high-dose group is to assess the pathogenicity of the vaccine to chickens. The antibody titers induced by the LX-OAI4S and the La Sota vaccination groups were almost identical in the low-dose group. The HI antibody titers reached 4.20 ± 0.75 and 4.30 ± 0.90 log_2_ at 7 dpv, respectively, both of which were significantly higher than those in the VG/GA group (Figure 5a). The HI antibody titers reached 5.60 ± 0.66 and 5.40 ± 0.80 log_2_ at 14 dpv, respectively, both significantly higher than those in the VG/GA group (Figure 5b). In the high-dose vaccination group, there were no significant differences in HI antibody titers at 7 dpv and 14 dpv (Figure 5d,e). After vaccination, the body weight of all chickens showed a trend in growth. Compared to the PBS control group, the weight gain of chickens in all vaccinated groups slowed down. At the two dosages, there was no significant difference in weight gain among the different vaccinated groups (Figure 5c,f). At the dose of 10 × 10^6^ EID_50_, the weight gain trend in the LX-OAI4S and VG/GA group was almost identical, and both were higher than that in the La Sota group. From day 9 post-vaccination, the LX group was significantly higher than the La Sota group, and this continued until the end of the observation period.

By observing the clinical signs after vaccination, at the high dose of 10 × 10^6^ EID_50_/0.1 mL, 5/10 chickens in the La Sota group showed respiratory symptoms with significant respiratory sounds. The chickens in LX-OAI4S and VG/GA groups showed no clinical signs (Table 4). No lesions were found in the proventriculus and duodenum of the chickens in all vaccination groups. In the LX-OAI4S group, mild hemorrhaging occurred in the throat of four chickens; the La Sota group had the highest number of chickens with throat hemorrhaging with eight cases, three of which were severe; the VG/GA group had throat hemorrhaging in four chickens, but only one was considered severe (Table 4).

To evaluate the protective efficacy against NDV challenge following ND vaccine administration via the spray route, chickens that had been vaccinated at a dose of 2 × 10^6^ EID_50_ were subjected to a challenge experiment. The LX-OAI4S group and the VG/GA group showed no clinical signs during the observation period, with a 100% immunoprotection rate for both groups. No virus shedding was detected in the oral cavities and cloaca of the LX-OAI4S group and the VG/GA group; the La Sota group did not show any virus shedding in the throat at 3 dpc. No virus shedding was detected in the throat at 5 and 7 dpc, but shedding was detected in the cloaca (1/10), with a shedding rate of 10% (Table 5).

### 3.4. Protective Efficacy of Recombinant LX-OAI4S Vaccine via Spray Route in Commercial Chickens

**Figure 6:**
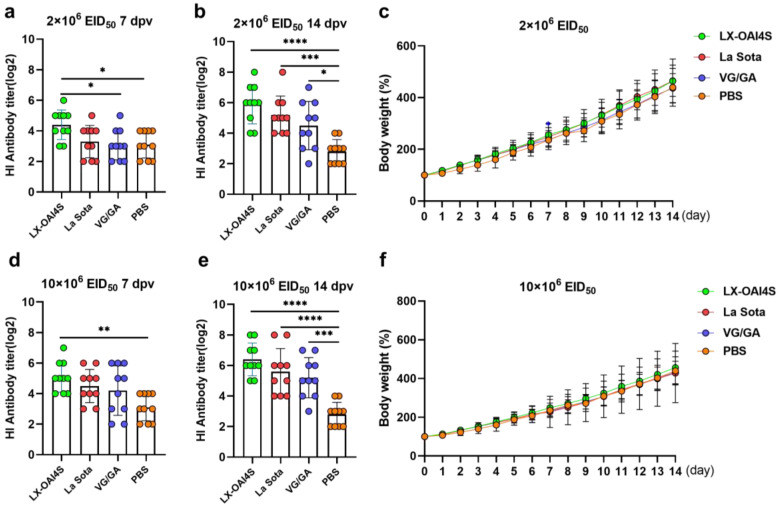
The HI antibody response and body weight changes in commercial chickens vaccinated with the ND vaccine via the spray route. One-day-old commercial chickens were randomly divided into four groups (*n* = 10) and vaccinated with recombinant virus strain LX-OAI4S, La Sota, and the VG/GA via spray route, respectively. The PBS control group was also established. Two vaccination doses of the vaccines were used, 2 × 10^6^ EID_50_/0.1 mL (**a**,**b**) and 10 × 10^6^ EID_50_/0.1 mL (**d**,**e**). The status of each chicken was observed after vaccination, and blood samples were collected on 7 and 14 days post-vaccination (dpv) to separate serum. The HI antibodies were then measured using the corresponding vaccine strains. Statistical analyses were performed by one-way ANOVA. * *p* < 0.05; ** *p* < 0.01; *** *p* < 0.001; **** *p* < 0.0001. The weight of chickens was continuously detected for 14 days after vaccination (**c**,**f**). The *p*-values shown for the comparison between the vaccinated group and the PBS group. Statistical analyses were performed by multiple *t*-tests. * *p* < 0.05; ** *p* < 0.01; *** *p* < 0.001; **** *p* < 0.0001.

We further evaluated the immunoprotective effect of LX-OAI4S vaccinated via the spray route in commercial chickens. The commercial vaccines VG/GA and La Sota served as positive control groups. Two vaccination doses of the vaccines were used, 2 × 10^6^ EID_50_/0.1 mL and 10 × 10^6^ EID_50_/0.1 mL. Commercial chickens possess high maternal antibody titers, with HI antibody titers against NDV reaching 3.1 ± 0.88 log_2_ (Figure 6a). At the low dose, the HI titer of LX-OAI4S reached 4.4 ± 0.97 log_2_ at 7 dpv, which was significantly higher than the PBS group and the VG/GA group (Figure 6a). There were no significant differences between the La Sota and VG/GA groups and the PBS group. At 14 dpv, the LX-OAI4S group was also higher than the La Sota and VG/GA groups but without significant differences (Figure 6b). At the high dose, the HI titer of LX-OAI4S was also higher than the La Sota and VG/GA groups at 7 and 14 dpv but without significant differences, with values of 5.1 ± 0.10 and 6.4 ± 1.07 log_2_, respectively (Figure 6d,e). At the two dosages, there was no significant difference in weight gain between the different vaccinated groups and the PBS group (Figure 6c,f). At the high dose, chickens in the LX-OAI4S and VG/GA groups did not show any clinical signs. However, in the La Sota group, one chicken exhibited symptoms of respiratory sounds and head shaking and died on the fifth day. No lesions were found in the throats and glandular stomachs of the chickens in all vaccinated groups. In the LX-OAI4S group, two chickens had mild hemorrhaging in the oral cavity; the La Sota group had six chickens with oral hemorrhaging, including one with severe hemorrhaging; and the VG/GA group had three chickens with throat hemorrhaging (Table 6).

At 14 dpv, the chickens were challenged with a virulent NDV strain. The chickens in the PBS group exhibited symptoms such as depression, reduced feed and water intake, or complete cessation of feeding and drinking. Chickens in the PBS group began to die from 4 dpc onward, with only two chickens surviving by 7 dpc, resulting in a mortality rate of 80%. Virus shedding was detected in both the throat and cloaca. All the chickens in the vaccinated groups did not show any clinical signs, there were no deaths, and they showed a certain inhibitory effect on virus shedding from the oral cavity and cloaca. In the La Sota and VG/GA groups, 5/10 and 4/10 chickens, respectively, showed virus shedding. In the LX-OAI4S group, only 2/10 chickens showed virus shedding, providing an 80% protection rate against virus shedding (Table 7).

### 3.5. Duration of LX-OAI4S Antibody Response Vaccinated via Spray Route

**Figure 7:**
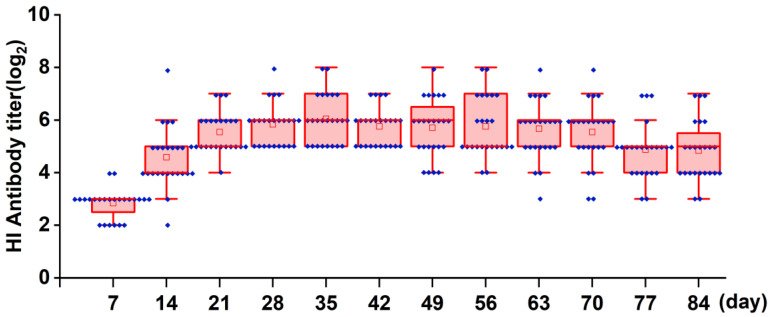
The sustained HI antibody response in chickens vaccinated with the ND vaccine via the spray route. One-day-old specific pathogen-free (SPF) chickens (n = 24) were vaccinated with the recombinant virus strain LX-OAI4S via the spray route at doses of 2 × 106 EID50/0.1 mL. The status of each chicken was observed after vaccination, and blood samples were collected every 7 days until day 84 to separate serum. The HI antibodies were then measured using the corresponding vaccine strains.

To evaluate the duration of antibody response, chickens vaccinated by spray with LX-OAI4S were continuously monitored for HI antibodies up to day 84. Throughout the prolonged observation period, the chickens vaccinated with LX-OAI4S did not exhibit any clinical signs such as respiratory issues, and their drinking and eating habits remained normal. The antibody titer showed rapid growth in the first three weeks post-immunization. By the third week, the average HI titer reached 5.54 ± 0.78 log_2_, and it reached its peak by the fifth week, with an average titer of 6.04 ± 0.96 log_2_. There was a slight fluctuation in antibody levels over the following six weeks but overall, the trend was a gradual decline. By the twelfth week, the average antibody titer was 4.83 ± 1.17 log_2_ (Figure 7).

## 4. Discussion

NDV poses a major global threat to the poultry industry, leading to significant economic impacts due to reduced egg production, slowed growth rates, and elevated mortality rates. This virus has the potential to infect a wide range of bird species, including chickens, turkeys, waterfowl, and so on, with its main transmission route being direct contact with infected birds through exposure to contaminated materials or airborne exposure [32]. ND presents with a variety of clinical signs, ranging from mild respiratory distress to severe neurological disorders and paralysis [33]. The epidemiological landscape of NDV has experienced substantial transformations in recent decades, marked by a rise in the frequency of outbreaks and the emergence of novel genotypes [2]. Notably, genotype VII.2 has emerged as a predominant strain in various regions, including Asia, Africa, and parts of Europe [4,5,6]. This particular genotype has been implicated in extensive outbreaks, presenting significant challenges for disease management and underscoring the imperative need for robust vaccination strategies and enhanced surveillance initiatives.

Vaccination is the primary means of controlling NDV. However, the presence of maternal antibodies (MDAs) can interfere with the immunological effect of live virus vaccines. Therefore, the development of NDV live virus vaccines that can overcome MDA interference is crucial for the prevention and control of NDV. In this study, we constructed a chimeric NDV live virus vaccine LX-OAI4S, which replaced the extracellular regions of the F and HN genes of the NDV LX strain with the corresponding regions of the F and HN genes of the A-VII vaccine strain. The HN protein recognizes the cell surface sialic acid receptor, and almost all the receptor activity sites and neuraminidase and antigenic sites are located in the extracellular region of the HN protein [34]. The isolation rate of HN protein E347K mutant strains of genotype VII NDV strains has been increasing year by year since around 2010 [35,36]. Our team has demonstrated that 347 is a critical determinant for the formation of the antigenic epitope (residues 345–353) on the HN protein [37]. The E347K mutation in the HN protein increases the antigenic difference between the widely used vaccine strain La Sota and the prevalent strains [28]. The HN protein of the A-VII vaccine strain also has the E347K mutation. The fusion protein (F) is also a surface glycoprotein that serves as the main antigenic epitope of NDV [38]. The interaction between the HN and F proteins plays an important role in virus fusion, and the residues in the intracellular region of the HN protein can mediate the F-HN interaction [39]. Studies have shown that when the F and HN genes of AMPV-2 are replaced in the corresponding regions of NDV virulent strains, the chimeric virus cannot be rescued successfully [40]. However, when the extracellular regions of F and HN are only replaced, the chimeric virus can be successfully obtained, indicating the importance of homologous intracellular and transmembrane regions for NDV replication [40]. Thus, we used genotype I NDV strain LX as a vaccine backbone, which is heterogenous to the circulating genotype VII NDV, to resist the interference of NDV maternal antibodies of genotype VII. Moreover, the substitution of the extracellular regions of the F and HN proteins of the vaccine strain A-VII not only ensure the effective rescue of the recombinant strain but also provide effective immunogenicity. The MDT and ICPI of LX-OAI4S both meet the standards for ND-attenuated strains, and the virus can maintain a high HA titer after continuous passage in chicken embryos. It can indicate that LX-OAI4S is an ideal vaccine strain with low virulence, genetic stability, good safety, and strong reproductive capacity in chicken embryos, which can be suitable for the immunization of young chicks.

To evaluate the immunoprotective efficacy of the recombinant LX-OAI4S vaccine, commercial vaccines La Sota and VG/GA were selected as controls. The LX-OAI4S group rapidly induced antibody production after vaccination, with HI antibody titers reaching 4.71 log_2_ at the early stage of administration, significantly higher than those of the two classical vaccine strains, meeting the criteria for complete clinical protection ≥ 4 log_2_ of HI titer [15]. This means that the recombinant LX-OAI4S vaccine can provide earlier protection against NDV for vaccinated chickens. After challenge with a virulent strain of genotype VII NDV, the LX-OAI4S vaccine group effectively inhibited virus shedding, indicating that the chimeric LX-OAI4S vaccine successfully induced immunoprotection against genotype VII NDV. In contrast, the two commercial vaccine strains La Sota and VG/GA showed virus shedding in the oral and cloaca at 3 dpc, fully validating that the NDV vaccine strains matched with the prevalent strain genotype can effectively prevent the shedding of NDV strong strains from the host [41,42]. Subsequently, we conducted an experiment to determine the minimum dosage of the recombinant vaccine LX-OAI4S. It was observed that the dosage of 10^5^ EID_50_ induced a more rapid HI antibody response at 7 dpv compared to the 10^4^ EID_50_ dosage, with no significant difference observed compared to the 10^6^ EID_50_ dosage. Both the 10^4^ EID_50_ and 10^5^ EID_50_ dosages of LX-OAI4S were effective in providing substantial protection against the highly virulent NDV.

This study aims to develop a novel vaccine that can be conveniently administered via spray and provides protection against the currently prevalent NDV genotype VII. Spray vaccination is an ideal choice for the mass immunization of poultry flocks, as it saves time and labor and reduces stress on the chickens. Therefore, we evaluated the immunoprotective effects of different doses of the recombinant ND vaccine LX-OAI4S in SPF chickens compared with the traditional vaccines VG/GA and La Sota. The results indicate that the LX-OAI4S vaccine administered via spray at both low (2 × 10^6^ EID_50_/0.1 mL) and high (10 × 10^6^ EID_50_/0.1 mL) doses induced high levels of HI antibodies in SPF chickens. Moreover, the LX-OAI4S vaccine provided 100% protection at the low dose, effectively preventing clinical disease and viral shedding. This is consistent with previous research demonstrating the robust immunoprotective effects of ND vaccines when administered via the spray route [18]. Spray immunization can effectively establish herd immunity against ND and reduce the incidence of the disease [19]. At the high dose, the LX-OAI4S vaccine group also showed good safety, with no obvious clinical signs or pathological changes observed. These findings suggest that the LX-OAI4S vaccine is a promising candidate for an ND vaccine and its administration via spray to SPF chickens can effectively prevent the occurrence of ND.

The immunoprotective effects of the LX-OAI4S vaccine were further evaluated and administered via spray to commercial chickens. The results indicate that the LX-OAI4S vaccine is capable of inducing high levels of HI antibodies in commercial chickens and providing 100% protection at a low dose, effectively inhibiting viral shedding (80%). At the high dose, no obvious clinical signs or pathological changes were observed in the LX-OAI4S vaccine group. These findings suggest that the LX-OAI4S vaccine can effectively overcome the interference of maternal antibodies, providing robust immunoprotection in commercial chickens. Maternal antibodies are a significant factor influencing the efficacy of live ND vaccines. How to surmount the interference of maternal antibodies has been a focal point of ND vaccine research. Research by Kapczynski et al. has shown that the in ovo application of antibody–antigen complex vaccines can effectively prevent NDV infection and reduce viral shedding in chickens with positive maternal antibodies [23]. Dimitrov et al. have constructed a recombinant vaccine by inserting an anti-sense sequence of avian interleukin 4 (IL4R) into the NDV vector, which effectively bypasses maternal antibodies and provides protection for the early prevention of ND [24]. Additionally, the administration of the three vaccines via spray in commercial chicken flocks had no impact on growth performance.

The duration of antibody response was also evaluated in SPF chickens vaccinated with the LX-OAI4S via the spray route. The results demonstrate that the LX-OAI4S vaccine is capable of inducing a durable HI antibody response in SPF chickens, with antibody levels remaining at a high level up to 12 weeks post-immunization. These findings indicate that the LX-OAI4S vaccine has a favorable immunological persistence and can effectively prevent the occurrence of ND. The duration of immunity is a crucial parameter for assessing vaccine efficacy. Similarly, research by Oberländer et al. has shown that live ND vaccines maintain sufficient seroconversion to protect flocks 12 weeks after vaccination [22].

The study demonstrated the successful development of a recombinant ND vaccine, LX-OAI4S, with enhanced immunogenicity against genotype VII NDV and the ability to overcome maternal antibody interference. The vaccine effectively induced robust immune responses and effectively suppressed viral shedding in both SPF and commercial chickens. Importantly, the spray administration of LX-OAI4S holds promise for convenient and efficient poultry vaccination, warranting further investigation for potential use in field trials and commercial settings.

## Figures and Tables

**Table 1:** Biological characteristics of LX-OAI4S virus.

	HAU	EID_50_	TCID_50_ (DF-1)	MDT	ICPI
P1	8.5	8.5	7.0	>120	0
P2	9.0	–	–	–	–
P3	9.0	–	–	–	–
P4	9.0	–	–	–	–
P5	9.0	8.83	7.5	>120	0
P6	9.0	–	–	–	–
P7	9.0	–	–	–	–
P8	9.0	–	–	–	–
P9	9.0	–	–	–	–
P10	9.0	9.0	7.5	>120	0

HAU: hemagglutination unit (log_2_); EID_50_: egg infective dose at 50% (log_10_/0.1 mL); MDT: mean death time (h); TCID50: tissue culture infective dose at 50% (log_10_/0.1 mL); ICPI: intracerebral pathogenicity index; P: passages; “–”represents not tested.

**Table 2:** Virus shedding in swabs from vaccinated groups.

Vaccine Strain	3 dpc		5 dpc		7 dpc	
	**O**	**C**	**O**	**C**	**O**	**C**
LX-OAI4S	0/7	0/7	0/7	0/7	0/7	0/7
La Sota	2/7	2/7	0/7	0/7	0/7	0/7
VG/GA	3/7	1/7	0/7	0/7	0/7	0/7
PBS	4/5	3/5	-	-	-	-

Chickens in vaccinated groups were challenged with 10^5^ EID_50_/0.1 mL of NDV strain JS2/06 via the intranasal and intraocular (IN/IO) route at 21 dpv. Oral (O) and cloacal (C) swabs were collected from chickens to evaluate viral shedding via virus recovery using SPF eggs at 3, 5, and 7 dpc. x/y: positive samples/total samples. “-” means the chicken is dead.

**Table 3:** Virus shedding in swabs from LX-OAI4S vaccinated groups with different doses.

Vaccine Dose	3 dpc		5 dpc		7 dpc	
	**O**	**C**	**O**	**C**	**O**	**C**
10^4^ EID_50_/0.1 mL	1/15	0/15	0/15	0/15	0/15	0/15
10^5^ EID_50_/0.1 mL,	1/15	0/15	0/15	0/15	0/15	0/15
10^6^ EID_50_/0.1 mL	0/15	0/15	0/15	0/15	0/15	0/15
PBS	13/15	11/15	-	-	-	-

Chickens in vaccinated groups were challenged with 10^6^ EID_50_/0.1mL of NDV strain JS2/06 via the IN/IO route at 21 dpv. Oral (O) and cloacal (C) swabs were collected from chickens to evaluate viral shedding via virus recovery using SPF eggs at 3, 5, and 7 dpc. x/y: positive samples/total samples. “-” means the chicken is dead.

**Table 4:** Clinical signs and histological change in vaccinated SPF chickens at a dose of 10×10^6^ EID_50_.

Chicken Number		1	2	3	4	5	6	7	8	9	10	Total
LX-OAI4S	clinical signs	-	-	-	-	-	-	-	-	-	-	0/10
	oral	-	-	+	+	-	-	-	+	-	+	4/10
	proventriculus	-	-	-	-	-	-	-	-	-	-	0/10
	duodenum	-	-	-	-	-	-	-	-	-	-	0/10
La Sota	clinical signs	-	+	-	-	+	+	-	+	+	-	5/10
	oral	+	++	++	+	+	-	+	+	++	-	8/10
	proventriculus	-	-	-	-	-	-	-	-	-	-	0/10
	duodenum	-	-	-	-	-	-	-	-	-	-	0/10
VG/GA	clinical signs	-	-	-	-	-	-	-	-	-	-	0/10
	oral	++	+	-	-	-	-	-	-	+	+	4/10
	proventriculus	-	-	-	-	-	-	-	-	-	-	0/10
	duodenum	-	-	-	-	-	-	-	-	-	-	0/10

Clinical signs: “+” indicates vaccinated chickens exhibited respiratory sounds or head shaking; “-” indicates no clinical signs. Oral, proventriculus, and duodenum: “-” indicates no lesions; “+” indicates mild hemorrhaging; “++” indicates severe hemorrhaging.

**Table 5:** Virus shedding in SPF chickens from different vaccinated groups at a dose of 2 × 10^6^ EID_50_.

Vaccination Strain	3 dpc		5 dpc		7 dpc	
	O	C	O	C	O	C
LX-OAI4S	0/10	0/10	0/10	0/10	0/10	0/10
La Sota	0/10	0/10	0/10	1/10	0/10	1/10
VG/GA	0/10	0/10	0/10	0/10	0/10	0/10
PBS	8/10	8/10	-	-	-	-

Chickens in vaccinated groups were challenged with 10^6^ EID_50_/0.1mL of NDV strain JS2/06 via the IN/IO route at 14 dpv. Oral (O) and cloacal (C) swabs were collected from chickens to evaluate viral shedding via virus recovery using SPF eggs at 3, 5, and 7 dpc. x/y: positive samples/total samples. “-” means the chicken is dead.

**Table 6:** Clinical signs and histological change in vaccinated commercial chickens at a dose of 10×10^6^ EID_50_.

Chicken Number		1	2	3	4	5	6	7	8	9	10	Total	Mortality
LX-OAI4S	clinical signs	-	-	-	-	-	-	-	-	-	-	0/10	0
	oral	-	-	+	+	-	+	-	-	-	-	2/10	
	proventriculus	-	-	-	-	-	-	-	-	-	-	0/10	
	duodenum	-	-	-	-	-	-	-	-	-	-	0/10	
La Sota	clinical signs	-	+	-	-	-	+	-	-	-	-	1/10	10%
	oral	+	+	-	++	-	death	-	+	+	+	6/9	
	proventriculus	-	-	-	-	-		-	-	-	-	0/9	
	duodenum	-	-	-	-	-		-	-	-	-	0/9	
VG/GA	clinical signs	-	-	-	-	-	-	-	-	-	-	0/10	0
	oral	-	-	+	-	-	+	+	-	-	+	3/10	
	proventriculus	-	-	-	-	-	-	-	-	-	-	0/10	
	duodenum	-	-	-	-	-	-	-	-	-	-	0/10	

Clinical signs: “+” indicates vaccinated chickens exhibited respiratory sounds or head shaking; “-” indicates no clinical signs. Oral, proventriculus, and duodenum: “-” indicates no lesions; “+” indicates mild hemorrhaging; “++” indicates severe hemorrhaging.

**Table 7:** Virus shedding in commercial chickens from different vaccinated groups at a dose of 2 × 10^6^ EID_50_.

Vaccination Strain	3 dpc		5 dpc		7 dpc	
	O	C	O	C	O	C
LX-OAI4S	2/10	0/10	1/10	0/10	0/10	0/10
La Sota	5/10	2/10	0/10	1/10	0/10	1/10
VG/GA	4/10	1/10	0/10	0/10	0/10	0/10
PBS	9/10	6/10	8/8	4/8	2/2	2/2

Chickens in vaccinated groups were challenged with 10^6^ EID_50_/0.1mL of NDV strain JS2/06 via the IN/IO route at 21 dpv. Oral (O) and cloacal (C) swabs were collected from chickens to evaluate viral shedding via virus recovery using SPF eggs at 3, 5, and 7 dpc. x/y: positive samples/total samples.

## Data Availability

The original contributions presented in this study are included in the article. Further inquiries can be directed to the corresponding author(s).
